# Stable Modality-Specific Activity Flows As Reflected by the Neuroenergetic Approach to the fMRI Weighted Maps

**DOI:** 10.1371/journal.pone.0033462

**Published:** 2012-03-14

**Authors:** Kuzma Strelnikov, Pascal Barone

**Affiliations:** 1 Hopital Purpan, Toulouse, France; 2 CNRS, UMR 5549, Faculté de Médecine de Rangueil, Toulouse, France; Institute of Automation, Chinese Academy of Sciences, China

## Abstract

This article uses the ideas of neuroenergetic and neural field theories to detect stimulation-driven energy flows in the brain during face and auditory word processing. In this analysis, energy flows are thought to create the stable gradients of the fMRI weighted summary images. The sources, from which activity spreads in the brain during face processing, were detected in the occipital cortex. The following direction of energy flows in the frontal cortex was described: the right inferior frontal = >the left inferior frontal = >the triangular part of the left inferior frontal cortex = >the left operculum. In the left operculum, a localized circuit was described. For auditory word processing, the sources of activity flows were detected bilaterally in the middle superior temporal regions, they were also detected in the left posterior superior temporal cortex. Thus, neuroenergetic assumptions may give a novel perspective for the analysis of neuroimaging data.

## Introduction

Dynamical neural field theories [1], [2] provide a theoretical framework to describe macroscopically the activity of the neuron ensemble. The need for the macroscopic approach stems from the fact that at the microscopic level even with only three states of activity for a cortical neuron (rest, activation, deactivation), approximately 10^37^ different configurations of cortical activity exist [1]; indeed, the precise analysis of each of them is beyond any computational facilities. Neural field modelling applies the equations from statistical mechanics and non-equilibrium thermodynamics to brain processes. The other direction of statistical and thermodynamic modelling of brain function is based on the free energy minimisation principle [3] and gradient reduction [4]. Since these models present a certain reflection of brain processes, it is possible to apply their principles to the other reflection of brain processes, which is observed in neuroimaging methods.

Current neuroimaging methods use different approaches to detect the changes in brain energy: the increase of metabolism reflected by the increase of blood flow, blood oxygenation (PET with H_2_O^15^ and BOLD fMRI), the increase in the metabolic rate of glucose (PET with FDG) [5]. fMRI measures the consumption of energy by the brain through oxygen consumption, which is needed for the synthesis of the energy-carrying ATP molecules. This energy is then used for different processes, resulting in changes of the electric field. Therefore the internal energy of molecules in a population of neural cells is primary, and is then used for the electric signalling. The link of the BOLD signal with electric measurements in the brain can reach up to 0.9 of correlation value [6], [7], so the measured energy consumption is closely coupled with electric activity. Actually, whether we measure electromagnetic or metabolic energy in the brain, these are two sides of the same coin [8], [9]. Combining the approaches of statistical physics and Bayesian hierarchical modelling of brain function, Friston [3], [10] proposed that the most suitable form of energy to describe brain mechanisms is free energy, and introduced the free energy minimization principle for the brain. Each molecule moves and has a potential to move, and thus each molecule has kinetic and potential energy. The sum of these energies for a brain volume is called internal energy. Free energy is a sort of internal energy with some corrections for temperature and entropy. This approach does not consider individual molecules and cells in the brain. Instead, it considers the brain as a field of energy with a certain mean value of energy in each small volume. We do not know the size of the smallest information-encoding volume in the brain; it may be infinitesimal. For practical purposes, the size of these brain volumes (voxels) can be arbitrarily chosen on the basis of the technically available spatial resolution and the need for precision.

When a neural signal reaches a neuroglial population, it induces changes in the states of many molecules in this population (receptors, mediators, enzymes, ions etc). Thus, it increases the internal energy and the free energy of this neuroglial population. The resulting activity can propagate to the neighbouring brain areas but it does not propagate backwards to the source of the input, i.e. in the antidromic direction. Thus, an abrupt change of neural activity and the related change of the BOLD signal appear in the direction of the input. Spontaneously, this abrupt change (the gradient) should disappear [11], [12] with time as it happens with temperature gradients. We apply the term “energy flow” [12] to the activity, which propagates in the same direction as the resulting abrupt changes (gradients) of activity. Energy flows in the brain were earlier defined as coherent spatial and temporal changes in the energy turnover of neuroglial units related to information treatment [13]; these flows are the result of the stimulation-driven transformations of energy that propagate in certain directions along the cellular structures (axons, dendrites, synapses, etc.). Energy flow is physiologically equivalent to activity propagation in the brain. E.g., if during visual processing information propagates from the thalamus to the visual cortex and then to the temporal cortex, this is the route of activity propagation or energy flow at the macroscopic level. The gradient vector indicates the direction of the fastest increase in the three-dimensional space. The fastest spatial increase occurs in the places where there is a burst of activity in a specialized neural population and a smaller activity in the input pathway downstream [14], [15]. Thus, for the spatial blob of visual activation the fastest activity increase would be at its “edge” receiving information from the thalamus. Localization of this abrupt change of activity permits to deduce the direction of signal propagation. As this abrupt change occurs every time for the same stimulus, it may be possible to detect it in the time-averaged spatial image of brain activity. This conclusion is supported by the linear relation between the increase in the number of neuronal spikes and the increase of the BOLD signal [16], [17].

**Figure 1:**
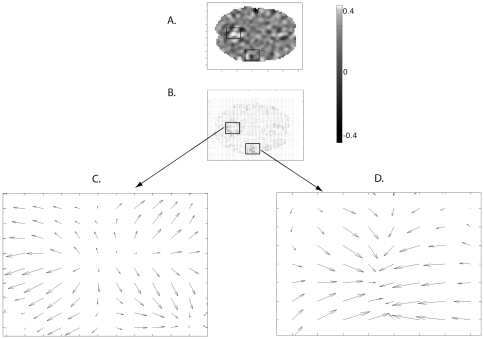
Illustration of divergence and gradients in a single brain slice of one subject during face processing. (A) The levels of divergence in a brain slice as coded by the white-black scale. (B) Each voxel in the slice is presented by an arrow – the direction of the arrow reflects the direction of the fastest change of the signal, the size of the arrow reflects the size of this change. These arrows are gradient vectors in each voxel. (C) The magnified part of B. where gradient vectors diverge. (D) The magnified part of B. where gradient vectors converge.

In this present study, we applied vector analysis to the fMRI data (Figure 1). In particular, we used statistical parametric mapping (SPM) to describe gradient vectors and their divergences in the fMRI data at the group level. As the BOLD signal reflects neuroglial activity, these gradients and divergences reflect gradients and divergences of activity in the brain. Compared with the classical activation analysis of fMRI data, which simply indicates where energy turnover is higher, gradients and divergences of the signal help understand the directions of energy flow (i.e., activity propagation) in the brain. The analysis of divergences should not be confused with classical activation analysis, because within the classical activations, which reflect a “plateau” of energy turnover, no divergence may exist; on the contrary, it may exist in the other regions. Positive divergence indicates the regions with the highest flow of energy outside of the region, i.e. sources of energy flows.

The challenge of this approach is to deduce the stimulus-related stable energy flows in the brain from the summary static images of the corresponding brain activity.

Firstly, we verify whether statistically significant gradients and divergences in the weighted contrast maps of the BOLD signal can be detected. As the answer is positive, in the Discussion section we analyse the neurocognitive interpretation of the results.

We applied the analysis to the exemplary face processing data of Henson et al. [19], available in free access on the Internet. The results and the logic of the interpretation are explained in detail for this data. The second analysis is done on a passive word perception dataset from a study performed in our laboratory [18]. The objective is to confirm that the sources of energy flows depend on sensory modality, so only the results for the sources are presented for this dataset given the spatial limitations of the article.

## Results

First of all, we applied our analysis of energy flows to the face processing data set. In the analysis of brain activations for the main effect of faces, significant activations where found bilaterally in the frontal and pre-central cortex, in the left inferior parietal cortex and in the right lingual region (Table 1).

**Table 1:** Brain activations for the main effect of faces.

Anatomic location	p(cor) cluster	cluster size	z-value	x	y	z
R cerebellum	0.000	7456	6.47	42	−48	−30
L cerebellum				−30	−60	−27
R lingual				6	−84	−3
L precentral, sup.	0.000	483	5.81	−48	12	33
L frontal, mid.				−42	51	12
L frontal inferior, operculum			−42	12	18	
R sup. motor area	0.000	814	5.44	3	15	45
L frontal sup. medial				−9	15	42
L inf. parietal	0.000	94	4.63	−33	−54	42
R middle cingulum	0.000	83	4.57	3	−18	24
R frontal sup.	0.013	44	4.26	36	60	12
L prefrontal sup.	0.025	38	4.08	−33	0	51

**Figure 2:**
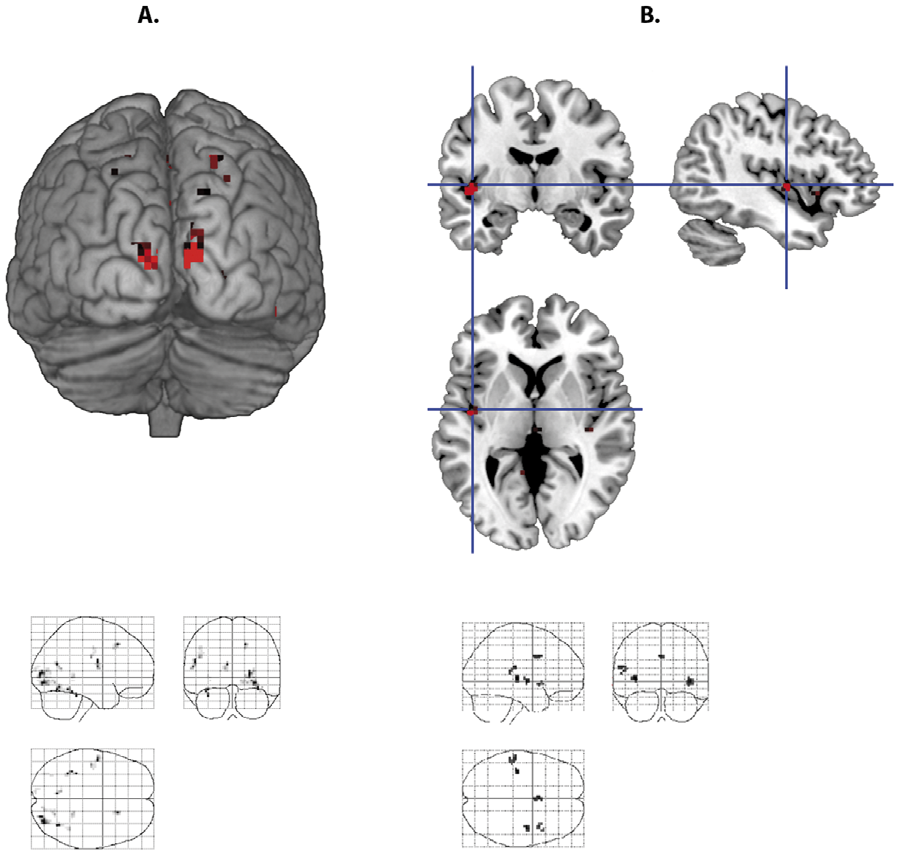
Divergence (sources) of the gradient vectors for face and word processing. (A) Sources for face processing in the occipital cortex. (B) Sources for auditory word processing in the superior temporal cortex.

The divergence analysis corresponds to the sources of energy flows, i.e. regions with the net flow of energy outside of the region. Divergence for face processing was positive in the right superior frontal region, in the left postcentral region, in the right middle occipital and in the fusiform regions bilaterally, in the left calcarine and right lingual regions (Table 2, Figure 2A). Negative divergence (convergence) for face processing was observed in the left thalamus and middle cingulum, in the left superior frontal and left postcentral regions, and bilaterally in the orbital parts of the frontal cortex (Table 2).

**Table 2:** Divergences for face and word processing.

Anatomic location	p(cor) cluster	cluster size	z-value	x	y	z
***Face, positive divergence (sources)***						
R fusiform	0.000	65	4.68	30	−84	−6
R mid occipital			4.68	24	−87	3
			3.79	30	−78	9
L fusiform	0.001	16	4.56	−33	−36	−24
			4.50	−33	−48	−15
R fusiform	0.000	21	4.49	36	−63	−15
			3.69	42	−45	−12
L postcentral, inf	0.000	21	4.42	−51	−6	21
R sup frontal	0.004	14	4.42	18	21	45
L calcarine	0.002	15	4.29	−6	−63	18
			3.83	−9	−57	12
L postcentral, mid	0.000	20	3.95	−45	−15	27
			3.41	−45	−12	39
			3.34	−39	−12	33
R lingual	0.002	15	3.81	21	−66	−6
			3.62	15	−81	−6
			3.28	15	−72	−6
R precuneus	0.001	16	3.71	21	−42	0
L calcarine	0.016	11	3.45	−9	−93	12
L mid occipital	0.026	10	3.31	−33	−81	0
**Face, negative divergences (convergences)**						
L thalamus	0.000	30	4.71	−12	−18	3
L middle cingulum	0.000	32	4.63	−3	−21	24
L inf frontal, orbital	0.016	11	4.34	−30	21	−12
R inf frontal, orbital	0.000	34	4.29	36	21	−9
L sup frontal, medial	0.000	22	4.22	−3	15	42
L postcentral, inf	0.006	13	4.02	−57	−15	15
***Word, positive divergences (sources)***						
L insula/Sup temporal	0.007	15	5.55	−36	−21	3
R insula/Sup temporal	0.005	16	5.26	42	−9	0
	0.014	13	4.67	0	9	36
L sup temporal/Inf parietal	0.003	17	4.45	−57	−24	21

Brain activity can induce different directions of gradient vectors, but for convenience of description it is useful to consider the significant projections of these vectors on the three principal axes. In the MNI and Talairach conventions of brain coordinates, the X axis goes from left to right, the Y axis goes from back to front and the Z axis goes upwards. Thus, positive projections on an axis in a given region imply that the direction of most energy changes in this region corresponds to the direction of the axis. Negative projections mean that the directions of energy changes in this region are mostly opposite to the direction of the axis.

For example, positive projections on the X axis mean that in these clusters, the more rapid changes of the BOLD signal happen in the “left-right” direction. It means that the predominant direction of energy flows in these clusters is from left to right.

**Figure 3:**
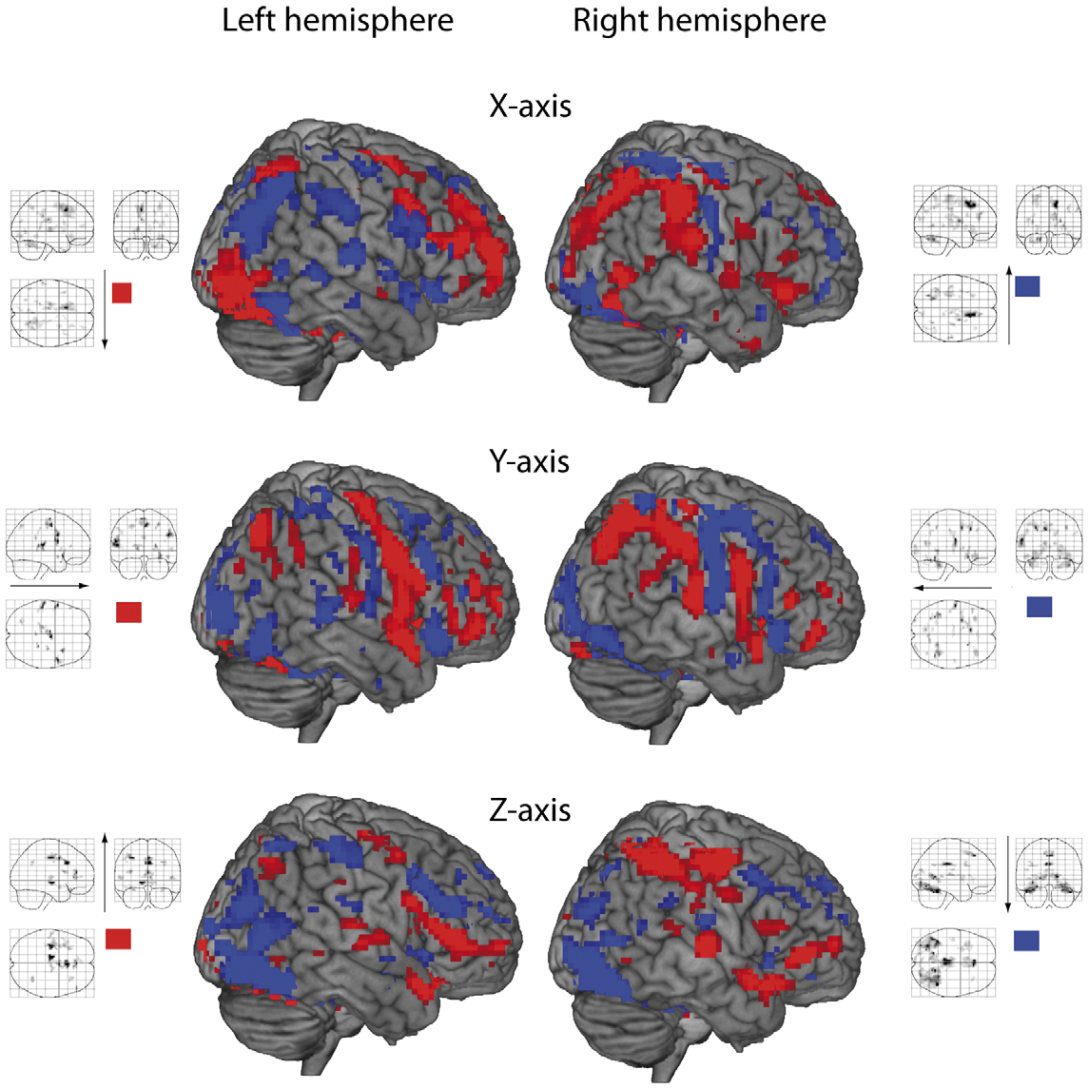
Projections of the gradient vectors on the X, Y and Z axes for face processing. Positive projections are indicated in red and negative projections in blue (p(uncor)<0.001 for illustration purposes).

Positive projections (the left-right direction) of the gradient on the X axis were found in the thalamus and cingulum bilaterally, in the left lingual cortex, in the right cerebellum and right inferior parietal cortex, in the bilateral frontal regions, in the left hippocampus and in the left supramarginal cortex (Table 3, clusters are ranged in the direction of the increase of the X coordinate; Figure 3). Negative projections (the right-left direction) of the gradient on the X axis were observed in the right superior frontal and left triangular frontal cortex, in the right putamen, in the right cingulum, in the right surpramarginal and angular regions, in the left superior temporal, left precentral and superior parietal regions, in the right angular and in the right fusiform cortices, and bilaterally in the cuneus (Table 3, clusters are ranged in the direction of the decrease of the X coordinate; Figure 3).

**Table 3:** Projections of the gradients for face processing, X axis.

Anatomic location	p(cor) cluster	cluster size	z-value	x	y	z
**X axis**, positive projections						
L supramarginal	0.000	38	4.61	**−60**	−33	30
L lingual	0.046	16	4.44	**−24**	−69	−3
L hippocampus	0.001	31	4.30	**−18**	−39	6
L thalamus	0.000	52	4.55	**−18**	−18	3
L cingulate, middle	0.000	37	4.83	**−9**	−21	24
L sup frontal, medial	0.000	338	5.68	**−9**	15	42
Cerebellum, culmen	0.000	43	4.29	**0**	−54	−24
R thalamus	0.004	26	3.73	**6**	−3	12
R cerebellum	0.000	131	4.83	**18**	−60	−30
R cingulate, post	0.016	20	3.68	**18**	−36	33
R caudate	0.012	21	3.91	**18**	−24	21
R middle frontal	0.003	27	4.29	**27**	0	51
R inf frontal, orb	0.000	40	3.89	**27**	30	−18
R parietal inf	0.004	25	4.25	**30**	−54	48
R sup frontal	0.035	17	3.80	**30**	51	12
**X axis**, negative projections						
R supramarginal	0.001	33	4.43	**60**	−21	36
R fusiform	0.012	21	3.49	**51**	−57	−18
R angular	0.000	44	4.19	**39**	−57	36
R putamen	0.001	32	3.98	**33**	−15	−3
R putamen	0.000	47	4.81	**24**	−3	12
R frontal sup medial	0.000	254	5.44	**15**	24	42
R sup frontal	0.000	46	4.53	**12**	39	27
R cingulum, middle	0.000	60	4.17	**9**	−21	24
R cuneus	0.035	17	4.30	**9**	−87	15
L cuneus	0.000	134	4.78	**−18**	−60	−24
L sup parietal	0.000	53	4.00	**−21**	−57	60
L inf frontal, triang	0.000	44	4.13	**−24**	21	−12
L precentral	0.000	165	4.36	**−24**	−24	63
L sup temporal	0.002	29	4.24	**−42**	−24	15

Clusters are ranged in the direction of the *increase* of the X coordinate for the positive projections and of the *decrease* of the X coordinate for the negative projections of the gradient (the corresponding columns highlighted).

Positive projections (the occipito-frontal direction) of the gradient on the Y axis were detected in the left precentral and superior temporal cortex, in the left thalamus in the right supplementary motor cortex, in the right hippocampus, and right precentral and inferior frontal regions (Table 4, clusters are ranged in the direction of the increase of the Y coordinate; Figure 3). Negative projections (the fronto-occipital direction) of the gradient on the axis Y were observed in the right occipital and orbital frontal regions, in the left superior and orbital frontal regions, in the left postcentral, in the left fusiform cortex, and in the left thalamus and in the left putamen (Table 4, clusters are ranged in the direction of the decrease of the Y coordinate; Figure 3).

**Table 4:** Projections of the gradients for face processing, Y axis.

Anatomic location	p(cor) cluster	cluster size	z-value	x	y	z
**Y axis**, positive projections						
R hippocampus	0.004	24	4.33	24	**−33**	9
L sup temporal	0.000	59	4.97	−54	**−24**	12
L thalamus	0.000	52	4.18	−12	**−24**	3
R precentral, sup	0.006	22	4.11	30	**−15**	51
R suppl motor	0.000	101	4.95	6	**−6**	51
L precentral, middle	0.000	54	4.78	−57	**6**	21
R precentral, sup	0.000	65	4.70	51	**6**	33
R Inf frontal, orb	0.000	34	4.36	33	**15**	−21
**Y axis**, negative projections						
R inf frontal, orb	0.000	82	4.20	36	**33**	−12
L sup frontal medial	0.006	22	3.73	0	**33**	42
L inf frontal, orb	0.000	52	4.23	−30	**30**	−3
L inf frontal, orb	0.005	23	4.09	−45	**24**	−9
L sup frontal medial	0.000	70	4.89	−6	**21**	42
R rectus	0.005	23	4.75	21	**15**	−12
L putamen	0.004	24	4.10	−15	**15**	−3
L middle frontal	0.000	89	4.99	−27	**3**	45
L postcentral	0.000	131	5.02	−57	**−9**	21
L thalamus	0.001	28	3.88	−12	**−12**	3
R precuneus	0.000	37	4.53	21	**−45**	0
R fusiform	0.000	143	4.78	42	**−51**	−18
L fusiform	0.000	120	5.44	−33	**−51**	−12
R precuneus	0.034	16	3.91	15	**−60**	36
R precuneus	0.045	15	4.13	3	**−66**	54
L calcarine	0.000	36	4.40	−9	**−69**	15
L cerebellum	0.025	17	3.59	−6	**−72**	−24
R sup occipital	0.000	53	3.96	24	**−93**	9

Clusters are ranged in the direction of the *increase* of the Y coordinate for the positive projections and of the *decrease* of the Y coordinate for the negative projections of the gradient (the corresponding columns highlighted).

Positive projections (down-up direction) of the gradient on the Z axis were found in the bilateral frontal orbital cortex, in the left subthalamic nucleus, in the left frontal triangular and opercular cortex, in the left superior temporal cortex, in the left and right cingulum, in the right supramarginal region and in the left precentral cortex (Table 5, clusters are ranged in the direction of the increase of the Z coordinate; Figure 3). Negative projections (up-down direction) of the gradient on the Z axis were in the left superior frontal and inferior opercular regions, in the left occipital cortex, in the left cingulum, thalamus, putamen, in the right lingual and inferior temporal regions (Table 5, clusters are ranged in the direction of the decrease of the Z coordinate; Figure 3).

**Table 5:** Projections of the gradients for face processing, Z axis.

Anatomic location	p(cor) cluster	cluster size	z-value	x	y	z
**Z axis**, positive projections						
L inf frontal, orbit	0.002	30	3.91	−27	21	**−18**
R inf frontal, orbit	0.011	22	3.66	30	27	**−18**
L subthamic nucleus	0.000	69	4.72	−12	−15	**−3**
Red nucleus			3.77	6	−15	**−3**
R inf frontal, triang	0.207	11	3.95	48	36	**6**
L sup temporal	0.002	30	3.76	−57	−15	**6**
L ant cingulum	0.000	89	4.45	0	36	**15**
L inf frontal, operc	0.004	27	3.94	−33	12	**18**
R middle cingulum	0.000	106	4.57	6	18	**33**
R supramarginal	0.011	22	3.90	30	−54	**36**
L precentral,sup	0.000	96	4.68	−30	−15	**45**
**Z axis**, negative projections						
L sup frontal, medial	0.000	123	5.05	0	27	**48**
L middle cingulum	0.000	117	4.87	−3	−30	**30**
L inf frontal, operc	0.000	36	3.96	−33	12	**27**
L cuneus	0.001	33	3.98	−3	−78	**18**
L thalamus	0.000	64	4.14	−6	−15	**9**
L putamen	0.002	30	3.84	−27	−3	**9**
R lingual	0.000	102	4.83	15	−51	**6**
L calcarine	0.015	21	3.96	−6	−93	**6**
L calcarine	0.000	48	4.66	−18	−54	**3**
L fusiform	0.000	398	5.21	−42	−45	**−21**
R inf temporal, post	0.000	513	4.79	45	−45	**−24**

Clusters are ranged in the direction of the *increase* of the Z coordinate for the positive projections and of the *decrease* of the Z coordinate for the negative projections of the gradient (the corresponding columns highlighted).

These results statistically confirm the existence of the gradients of brain activity in response to the given cognitive load. The gradients are supposed to reflect the direction of energy flows (i.e., activity propagation, see the biophysical interpretation below). Thus, our analysis suggests that there are significant projections of energy flows on the major axes X, Y, Z both in the positive and negative directions of the axes.

Concerning the auditory word discrimination task, we conducted only the analysis of the sources of energy flows (positive divergence of gradient vectors). For word processing, positive divergence was detected in the insula and middle superior temporal regions bilaterally and in the left posterior temporal region at the junction with the inferior parietal lobule (Table 2, Figure 2B).

## Discussion

As can be seen from comparing the results of activation analysis from Table 1 with the results for the analysis of divergence and gradient (Tables 2,3,4,5), the latter provide additional information on brain activity. This statistically significant additional information proves a potential importance of the proposed method to clarify the details about the distribution and dynamics of brain response, and in particular about the directions of energy flows in the brain. Besides, a potentially important output of this analysis is the localization of the sites in brain cortex with a significant divergence or convergence of energy flows.

Positive divergence (Table 2, Figure 2) indicates brain clusters where net flow of energy from the voxels is outside; these voxels and their clusters constitute a source of energy flows for the surrounding voxels. As the task was related to visual face processing, we confirmed using our approach that most of these sources are in the occipital cortex (BA 19). However, as face expressions also involve an internal representation of the sensory-motor component, other sources are found in the postcentral and superior frontal regions. These results are in agreement with previous results based on static analysis of brain activity [21], [22].

Further, the analysis of auditory word processing was realized to validate our methodological approach. Indeed, the sources of energy flows in this case were found in the vicinity of the auditory cortex bilaterally, and in the language-specific posterior superior temporal cortex on the left (Table 2). The results are in agreement with the data on auditory speech processing with classical activations [23], [24] and are totally differentiated from the results obtained by this method when applied to face processing.

If positive divergence indicates the sources of the flows of energy, where do these flows finally end up in the brain? The answer can be provided using the analysis of the negative divergence (convergence). This analysis (Table 2) indicates voxels in which the net flow of energy is in the inward direction; these voxels constitute the equivalent of “sinks” for energy flows. According to Table 2, these “sinks” can be detected in the orbital frontal cortex and in the middle cingulum. Interestingly, they are also found in the thalamus and in the postcentral region, which can be related to feedback circuits involving these regions [25].

The analysis of divergence provides information about the sources and “sinks” of energy flows in the brain but no information about the directions of these flows in the brain. The exact reconstruction of these directions would of course be very complicated, given the existence of various parallel pathways and feedback loops. Currently the spatial and temporal resolutions of the fMRI technic are ineffective to determine precisely such dynamic features. However, the analysis of gradients may indicate some favored directions of energy flows in the group of subjects for a certain task.

Concerning the face processing data from Table 3, the right-directed flow (“X axis, positive projections”) happens in cortical areas of the left hemisphere (the supramarginal and lingual regions, the thalamus) as well as in some right hemispheric regions (the middle frontal, the inferior frontal and parietal regions). Interestingly, the opposite flow (to the left) happens in a large set of brain structures (“X axis, negative projections”), including the temporal and frontal cortices of both hemispheres.

Another interesting finding concerns the left thalamus, in which the “left to right” flow is localized (“X axis, positive projections”). This may indicate a localized neural circuit in the thalamus.

Considering projections of the gradient on the Y axis (Table 4), one can see that the “backwards” direction of energy flows (negative projections) is present in many structures from the posterior part of the brain (the occipital region) to the anterior parts in the frontal regions. In between, these flows involve the thalamus and the postcentral cortex. This corresponds to the well known cortical loops for processing visual information, which is sent from the initial sources in the occipital regions (Table 2, Figure 2A) to the frontal cortex for the integrative analysis and then projected backwards for the top-down guidance of visual strategies [21]. The anterior-posterior directions in the occipital region can be also related to the fact that the thalamus is in a more anterior position relative to the occipital region.

Of interest is that our analysis of energy flows allows to get information of the following steps of information processing after it has reached the most anterior point in positive projections (y = 15 in “Y axis, positive projections”). The first line in “Y axis, negative projections” shows that the energy flow moves higher in the right frontal lobe (from z = −21 in “Y axis, positive projections” to z = −12 in “Y axis, negative projections”) and then continues a backward direction in a more posterior region on the left (with y = 30 and 24). Then it goes to the medial part of the left frontal lobe terminating in the left medial frontal cortex. Thus, this analysis permits us to reveal a complex loop for face processing in the frontal cortex.

An interesting observation concerns the most posterior parts of the brain (see the bottom of “Y axis, negative projections” in Table 4: the right superior occipital region at y = −93). As on the right this is the most posterior point, one could expect the flow forward from this point. The flow in the right precuneus, which is more anterior to the region in the right occipital region, is also directed backwards, from the presumable transfer in the corpus callosum.

Considering the Y projections, we have been able to reveal opposite flows in the left and right thalamic region in the same horizontal plane (at z = 3). One can see from “X axis, positive projections” in Table 3 that in this horizontal plane (at z = 3), there is a flow oriented in the “left-right” direction. To understand the spatial relation of these flows, let us consider the Y coordinate for each cluster in the left thalamus. In “Y axis, positive projections” (Table 4), which reflects the flow forward, the coordinate of the left thalamus is y = −24. In “Y axis, negative projections”, which reflects the flow backwards, the coordinate of the left thalamus is y = −12. Thus, the forward flow is localized more posteriorly than the backward flow. The “left-right” flow in the left thalamus (“X axis, positive projections” in Table 3) is between the forward and backward flows, at a coordinate of y = −18. This consideration permits us to suggest a circuit in the left thalamus, mostly existing in the horizontal plane.

Z axis projections in Table 5 indicate brain regions where the directions of energy flows are mostly in the upward and downward directions. Some of them are induced by anatomical constrains such as in the most inferior orbital parts of the frontal cortex, in which the direction of flows is mostly upward (“Z axis, positive projections”, first lines). The mostly upwards flow in the anterior cingulum suggests that it may go to the frontal cortex. This assumption is supported by the presence of bilateral flows in the frontal regions.

Interestingly, the flow in the left superior temporal region is directed upwards. The possible continuation of this flow may be indicated by the upward flow in the left inferior frontal region and in the left precentral region. The highest point (z = 48), from which the flows go in the downward direction, is in the medial part of the left superior frontal region (“Z axis, negative projections”). The downward direction is also significant in the occipital regions. It becomes clearer if we take into account that the inferior temporal region (z = −24) is situated lower in the brain than the occipital cortex - obviously, this is one of the targets of information and energy flows from the visual cortex.

Comparing “Z axis, positive projections” and “Z axis, negative projections”, one can notice that at the same coordinates x = −33 and y = 12, there are two opposite flows in the left operculum. One is higher, at z = 27 (“Z axis, negative projections”), and is directed downwards; the other is lower, at z = 18 (“Z axis, positive projections”), and is directed upwards. The existence of a circuit in the left operculum is probable. This rather local circuit may explain activation of this region in the analysis of activations (Table 1).

**Figure 4:**
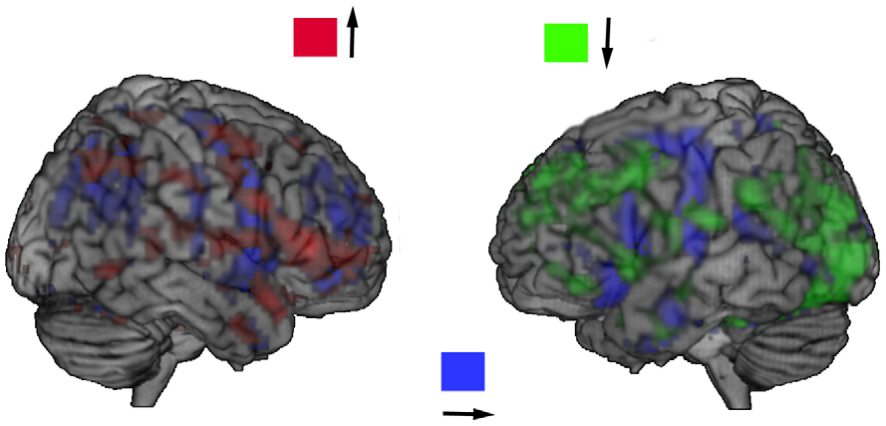
The left-right and top-down projections of the gradients, the intensity of projections coded by colours. The following direction of energy flows in the frontal cortex is detected: the right inferior frontal = >the left inferior frontal = >the triangular part of the left inferior frontal cortex = >the left operculum. The whole range of t-values is used for illustration purposes.

In the previous considerations of the gradients, we suggested the following direction of energy flows in the frontal cortex: “right inferior frontal – left inferior frontal – left medial frontal cortex.” (Figure 4). As the projection of the gradient in the triangular part of the left frontal cortex is to the left (“X axis, negative projections”), evidently, the flow goes next to the left operculum, where it enters the more localized circuit of processing. This direction of energy flows may clarify the long-standing debate between the holistic and analytical views on face processing (see [26] for the discussion); it suggests that the holistic processing in the right hemisphere is followed by the analytical processing in the left hemisphere.

Thus, the potential significance of our analysis consists in its ability to provide novel information on the cortical dynamics of face processing, which was unavailable in the classical activation analysis of the fMRI dataset [19]. The results of the analysis provided novel evidence for the favoured directions of the brain activity propagation during the given task including the inter-hemispheric relationship.

### Limitations of the method

This method proposes to detect stable gradients of the fMRI signal inside the grey matter volume. If a peak for a gradient projection falls at the border of white and grey matter, the observed gradient may be related to the difference in signal between white and grey matter. Firm conclusions can be based only on those peaks, which are inside grey matter; this should be controlled on the basis of the anatomical fMRI images. The gradient between extra-cerebral tissues and grey matter does not present a problem for contrast images because there are NaN values outside the brain, which are not used in Matlab calculations. However, it may present a problem for some other types of brain images with real values outside the brain; the values outside the brain should be verified.

For the same reasons, the gradients in the small nuclei (e.g., the left subthalamic nucleus in Table 5, “Z axis, positive projections”) surrounded by white matter should be interpreted with caution. One should also check that the considered peaks of the gradients are not situated in the walls of cerebral.

### Perspectives

Though there are technical and interpretational limitations to apply our method to the initial un-weighted and unprocessed images of the BOLD signal, the possibilities of this approach should be explored. One constraint to solve consists in the non-functional gradients related to the extra-cerebral tissues in the initial images. Another constraint is the possible negative influence of smoothing on gradient images. Smoothing is helpful in the present analysis because it enhances the overlap in the spatially varying areas within one subject, creating the gradient in the vicinity of this overlap in the summary weighted images. However, the influence of smoothing, when applied to the initial gradient images, is questionable and should be separately explored.

Another perspective is related to the development of the statistical methods based on the present method. For example, correlations of behavioural scores with the intensity of energy flows in some directions may be an interesting approach. The region of interest analysis can help determine the major directions of the input or output from the given area. A more complex analysis with the same statistical ideology could be the “path of interest” analysis, in which the direction of energy flows is estimated along the given path in the cortex basing on the literature data on cortical connections. As brain activity at rest reflects long-term adaptive strategies [27], in addition to the effects of stimulation it would be interesting to develop the applications of this method to the resting-state activity.

### Conclusions

In addition to the classical analysis of brain activation, the implementation of the vector analysis into the analysis of neuroimaging data during face processing provides important insights into the understanding the information-driven propagation of energy flows between and within activated areas. The sources, from which activity spreads in the brain during face processing, were detected in the occipital cortex as well as in the postcentral and superior frontal regions. The later regions correspond probably to the loci where the sensory-motor component of the face processing may be localized. Analysis of the energy flows suggested inter-hemispheric transfer between the visual and the frontal cortices. In addition, we have been able to demonstrate a complex loop in the frontal cortex that originates in the right inferior frontal cortex and ends in the triangular part of the left inferior frontal cortex. Further this path propagates backwards to the left operculum, where a localized circuit was described. Regions, which gather information from the other areas (“sinks” of energy flows) were localized in the orbital frontal and postcentral cortices as well as in the thalamus and in the middle cingulum.

We have confirmed that this method differentiates between the modalities of sensory processing that is important to validate the discussed physical theory at the neurocognitive level. For the auditory word processing, the sources of energy flows were detected in the middle superior temporal regions bilaterally and in the left posterior temporal region at the junction with the inferior parietal lobule, in accordance with classical activation studies. Thus, the proposed approach leads to reasonable results and presents an interesting perspective for the analysis of neuroimaging data based on biophysical assumptions.

Altogether, the visualization of energy flow based on the computational thermodynamic approach to static activation maps allows access to a dynamic view on the transfer of information in the brain. Such a method that can be applied to both sensory and cognitive functions is promising to reveal functional activity streams in normal and pathological brain. Further developments are needed to verify the validity of this method in comparison with activity flows reflected by electrophysiological measurements and on the simulated fMRI datasets. The consideration of the brain as energy field may be helpful in providing new insights into brain function, both at the theoretical and methodological levels.

## Materials and Methods

### Statistical and vector analysis

As fMRI data, we used a multi-subject event-related fMRI dataset from the SPM site (http://www.fil.ion.ucl.ac.uk/spm/data/face_rfx/). This data was collected by Henson et al. [19] in a study of face repetition effects. In this study, the subjects were repeatedly presented with various famous and non-famous faces; the subject pressed a key to detect famous faces and those seen previously during the session. The baseline was a chequerboard presented between the faces. The dataset comprises 12 subject-specific t-contrasts on the main effect of faces versus baseline on the canonical HRF (a contrast collapsing across face-types) as “con*.img” files.

A second dataset on auditory processing was included in this analysis and originated from our work on speech processing. Subjects lay in the camera with eyes closed and listened to disyllabic words. One out of 13 stimuli was randomly repeated, and the subjects were instructed to press the button when they heard a repetition. The dataset comprises 15 subject-specific t-contrasts on the main effect of words versus silent baseline on the canonical HRF as “con*.img” files.

To each image file from the dataset, the following Matlab procedure was applied (attention should be paid to the indicated switching of the X and Y axes between the SPM and Matlab conventions):

activity = load_nii(‘con_0006.img’); % load file

[GradientX,GradientY,GradientZ] = gradient (activity.img); % gradient calculation, its projections on the axes as output

activity.img = GradientX; save_nii(activity, [‘GradientY_0006.img’]); % save the image with projections of the gradient vector on the axis X (the same for the axes Y and Z, **switch Y and X!**)

[x y z] = meshgrid(1∶63,1∶53,1∶46); % meshgrid creation, the size of the fMRI images

div = divergence (x,y,z,GradientX,GradientY,GradientZ); % calculation of the divergence of the gradient vector

activity.img = div; save_nii(activity, [‘div_0006.img’]); % save the divergence image

We used a special toolbox NIFTI for loading SPM images into Matlab: http://www.rotman-baycrest.on.ca/~jimmy/NIfTI/

As a result, we had for each subject five images: the original one, three images with projections of the gradient vector on the axes X, Y, Z and one image with the divergence. For the word processing dataset, only the divergence procedure was applied.

For each type of the images, a one sample t-test was performed in SPM5 to estimate the images at the group level.

To find areas where divergence is positive or negative, contrasts [1] and [−1] were used. The same contrasts were used to find positive or negative projections of the gradient on the axes X, Y, Z. Clusters were considered significant at p<0.05, FWE corrected.

The classical SPM estimation consists in estimating the folowing regression-like equation:
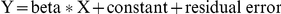
where Y is the observed value per voxel and X is the value predicted by our model (e.g, 1 for stimulation, 0 for the absence of stimulation). The t-value is generally defined by the relation beta/(the residual error) with the beta-value, which provides the best fit of the data. The t-value is then compared with a theoretical distribution to get a p-value. Given the large number of voxels, this p-value is then corrected by special procedures for multiple comparisons.

We use a one-sample t-test for our images of divergences and gradient projections to detect a mean value per voxel; our model is just a column of ones for X in the above equation [20]. In this case, our null hypothesis is beta = 0 and the alternative hypothesis is beta>0. If we want to test the other alternative hypothesis of beta<0, we simply multiply the beta-value in the equation by −1. In SPM, the first case of positive betas corresponds to the [1] contrast, the second case of negative betas to the [−1] contrast. The [1] contrast corresponds to the case when the X and Y go in the same direction (when X is positive, Y is positive). The [−1] contrast corresponds to the case when X and Y go in the opposite directions (when X is positive, Y is negative). As our X is always positive, the contrasts simply test whether the mean values of Y are positive or negative in a given voxel. It permits to say whether the mean divergence is significantly positive or negative or whether the mean values of the gradient vector projections are significantly positive or negative in each voxel.

The MRIcron software was used for illustrations (http://www.mccauslandcenter.sc.edu/mricro/mricron/index.html).

### Type of images for the analysis

The contrast images we used for the analysis represent spatially distributed images of the weighted for the variance sum of the parameter estimates for a particular contrast at each voxel. Thus a contrast image summarizes the activation effect for a particular subject. In particular, since the images we use for the analysis are the contrasts of sensory stimulation with baseline activity, we can claim that these changes in brain energy are related specifically to information processing and not to the other states of the brain. This would have been difficult to claim if we had not taken the images statistically weighted by the baseline. Besides, the initial images' highest gradients are related not to the brain activity but to the difference in signal between the brain and surrounding tissues.

Although energy flows in the brain are highly unstable over time, the stability of the flows means that they persist during stimulation, so that the temporal component can be left out of the analysis. This justifies the use of static images as a summary over the period of stimulation. In other terms, the temporal stability of the calculated gradients is ensured by the statistical stability of the estimates in the weighted contrast images. The proposed calculation is not a new type of statistical analysis but just a mathematical transformation of the data; it does not require validation by split-half or other methods. We use the traditional t-statistics implemented in SPM to estimate the transformed images at the group level.

### Biophysical interpretation

In this section, we will consider some very general physical ideas concerning the interpretation of the free energy gradients and energy flows. The detailed modeling is outside of the scope of the present article.

Suppose the macroscopic states of a thermodynamic system depend upon *r* extensive variables *Xi*, *i* = 1, *r*. There are then *r*+1 independent extensive variables consisting of the set *Xi* supplemented by entropy *S*. The fundamental relation for the system can be expressed in the form of internal energy *U* = *U*[*S*, *X*_1_, …, *Xr*].

In the brain and in many other systems, *Xi* can be replaced by the volume *V* and particle number *N*, and then U = *U*[*S*, *V*, *N*]. However, entropy *S* is not a convenient variable to measure experimentally, so by means of the Legendre transformation, the Helmholtz free energy *F* is introduced *F* = *F*[*T*, *V*, *N*] where T is absolute temperature.

Both internal energy and free energy are thermodynamic potentials, the meaning of which will be considered later.

If *F* depends on time t, the free energy minimization principle [3], [10] requires that the 1^st^ derivative of F with respect to time should tend to be zero and the 2^nd^ derivative to be positive. The 1^st^ derivative in the Cartesian coordinate system can be expressed like this:
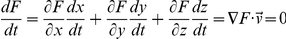
Here, the gradient of energy 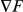 is generally in the same direction as the velocity of its flow 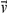 and their dot-product becomes zero because of the gradient destructive nature of self-organizing nonequilibrium living systems [11]. It follows that the gradient of *F* in these voxels tends to equal zero:
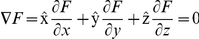
As our analysis of fMRI data suggests the existence of the gradients, which are stable in time, the minimization principle is counterbalanced by some forces, which are evidently related to information treatment.

In physics, the potential energy *U*(**r**) corresponding to a force **F**(**r**) can be expressed as an integral of **F**(**r**) where **r** is a vector pointing from the origin to the given location. The work W done by **F**(**r**) in a small displacement from **r** to **r**+*d* **r** is:
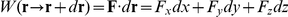
The work of the external force is positive when this force acts in the direction of energy increase (e.g., climbing the mountain), and thus equals the change of potential energy:
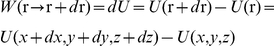
We can express this change of energy as the sum of changes along each axis x, y, z:
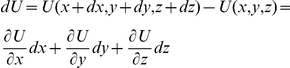
We can see that the same work of the external force can be expressed either as the function of the force or as the function of the potential field, against which this force works:
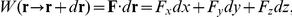


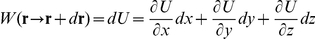



Given these two equations, the external force equals the gradient of *U*:
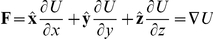
By analogy, if we consider the expression for the gradient of the free energy field *F*

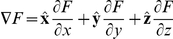
the negative value of this gradient corresponds to a certain internal “force”, which is directed “downhill” the free energy values. This is not a usual force but a certain generalization called thermodynamic force. As we have shown, this force would normally drive everything in a voxel down to the minimum of the free energy and the gradient will become zero. If the gradient remains constant, it means that some forces act in the same direction as the gradient of the field but opposite to the thermodynamic force. These are forces related to the transmission of the neural signal, and their direction is defined by the last equation for 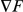. The nature of these forces is unknown; they may be related to the mechanisms that prevent neural activity from moving backwards along the same path.
